# Enhancement of Physical Characteristics of Styrene–Acrylonitrile Nanofiber Membranes Using Various Post-Treatments for Membrane Distillation

**DOI:** 10.3390/membranes11120969

**Published:** 2021-12-09

**Authors:** Reza Sallakhniknezhad, Manijeh Khorsi, Ali Sallakh Niknejad, Saeed Bazgir, Ali Kargari, Mohsen Sazegar, Mohsen Rasouli, Soryong Chae

**Affiliations:** 1 School of Mechanical Engineering, Beijing Institute of Technology, Beijing 100081, China; Rezaniki1990@gmail.com; 2 Department of Chemical Engineering, Science and Research Branch, Islamic Azad University, Tehran 1477893855, Iran; khorsi.manijeh@gmail.com; 3 Nano Polymer Research Laboratory (NPRL), Science and Research Branch, Islamic Azad University, Tehran 1477893855, Iran; aliniknejad1991@gmail.com; 4 Department of Polymer Engineering, Petroleum and Chemical Engineering Faculty, Science and Research Branch, Islamic Azad University, Tehran 1587856614, Iran; 5 Membrane Processes Research Laboratory (MPRL), Department of Chemical Engineering, Amirkabir University of Technology, Tehran 1591634311, Iran; kargari@aut.ac.ir; 6 Department of Polymer Engineering, Amirkabir University of Technology, Tehran 1591634311, Iran; Engineer.sazegar@gmail.com; 7 SEM Lab, Central Laboratory, Amirkabir University of Technology, Tehran 1591634311, Iran; rasouli33@gmail.com; 8 Department of Chemical and Environmental Engineering, University of Cincinnati, Cincinnati, OH 45221, USA

**Keywords:** styrene–acrylonitrile nanofiber membranes, post-treatment methods, mechanical strength, pore size distribution, membrane distillation

## Abstract

Insufficient mechanical strength and wide pore size distribution of nanofibrous membranes are the key hindrances for their concrete applications in membrane distillation. In this work, various post-treatment methods such as dilute solvent welding, vapor welding, and cold-/hot-pressing processes were used to enhance the physical properties of styrene–acrylonitrile (SAN) nanofiber membranes fabricated by the modified electrospinning process. The effects of injection rate of welding solution and a working distance during the welding process with air-assisted spraying on characteristics of SAN nanofiber membranes were investigated. The welding process was made less time-consuming by optimizing system parameters of the electroblowing process to simultaneously exploit residual solvents of fibers and hot solvent vapor to reduce exposure time. As a result, the welded SAN membranes showed considerable enhancement in mechanical robustness and membrane integrity with a negligible reduction in surface hydrophobicity. The hot-pressed SAN membranes obtained the highest mechanical strength and smallest mean pore size. The modified SAN membranes were used for the desalination of synthetic seawater in a direct contact membrane distillation (DCMD). As a result, it was found that the modified SAN membranes performed well (>99.9% removal of salts) for desalination of synthetic seawater (35 g/L NaCl) during 30 h operation without membrane wetting. The cold-/hot-pressing processes were able to improve mechanical strength and boost liquid entry pressure (LEP) of water. In contrast, the welding processes were preferred to increase membrane flexibility and permeation.

## 1. Introduction

As a consequence of rapid population growth, climate change, and destructive human activities, the accessibility of potable water has become a global issue. In addition, petrochemical, mining, steel, and other industries exacerbate the current situation even further [1,2,3].

Over the decades, membrane-based desalination technologies have been introduced as a viable solution to produce potable from various water sources, including seawater. For example, reverse osmosis (RO) is a well-known membrane process for seawater desalination due to its good performance (>99% removal of monovalent and multivalent ions). However, the high capital cost and excessive energy consumption to provide high pressure, membrane fouling, and brine disposal are reported as the main challenges of this process [4,5,6].

Membrane distillation (MD) is a promising method for producing pure water from saline water sources. In MD, the water vapors in the feed side pass through the pores of a hydrophobic membrane and turn into liquid on the permeate side. The required driving force for water vapor transportation is supplied by the temperature difference between feed and permeate sides, and the liquid evaporation occurs on the membrane surface [7,8,9,10,11,12,13]. Interestingly, there is no limitation on treating hypersaline brine, compared with RO [14], and MD also can use waste heat, renewable energies, and low-grade heat as energy sources of the process, providing desirable possibilities [15,16].

Nonetheless, the MD process is not fully developed at an industrial scale, mainly due to the lack of hydrophobic membranes with good wetting and fouling resistance [17]. Typically, a membrane having high porosity and hydrophobicity with superior mechanical properties is needed to demonstrate a promising performance in MD [18,19,20]. Non-solvent-induced phase separation (NIPS) and thermally induced phase separation (TIPS) are the most common approaches to fabricate membranes; however, low permeation and weak hydrophobicity impede their full application in MD [21].

The electrospinning process is a potential alternative method for fabricating highly porous membranes with high hydrophobicity, tunable pore size, and thickness, using high electrical charges to produce a non-woven network of nano/microfibers [22]. It is a fairly simple fabrication process, compared with the other membrane fabrication processes such as NIPS and TIPS [23,24]. While outstanding properties ascribe to nanofibrous membranes, these membranes riddle with larger pore size and lower mechanical properties than the membranes designed by casting methods [22,25,26,27]. Therefore, post-treatment processes are needed to address these challenges. Thus far, hot pressing [28] and chemical crosslinking [29] are the most used methods to lower pore size and enhance membrane robustness. However, crosslinkers are harmful to the environment and the human body. Surface coating is another method to reduce the pore size of the MD membranes. However, this method adversely affects membrane permeation by reducing effective porosity and pore size of membranes [30,31].

Recently, solvent vapor welding [32] and dilute solvent welding [33] have been adopted to increase mechanical strength and reduce the pore size of nanofibrous membranes. Nanofibrous samples are directly subjected to the solvent vapors to fuse fibers in their junction points in the vapor welding process. However, it is reported that it takes a longer fabrication time to be completed depending on the type of process [32,34]. Additionally, as the only solvent present in the air, it is hard to control the welding pace and construct a uniform fibrous structure since the solvent vapors can be absorbed totally by the surface rather than the inner structure.

Dilute solvent welding seems more practical than vapor welding as the non-solvent (i.e., ethanol) thoroughly wets the nanofibrous membrane. By applying heat, the solvent can fully weld the entire structure. It needs to be stated that the welding solution of this process is composed of wetting liquid (major component) and solvent (minor component) to cause welding by only heating the sample.

In this study, nanofibrous styrene–acrylonitrile (SAN) membranes were fabricated through the modified electrospinning process, electroblowing, and using a low-toxic dimethyl sulfoxide (DMSO) solvent to improve low productivity [35,36,37,38]. Then, cold-pressing, hot-pressing, vapor welding, and dilute solvent welding processes were used to enhance the physical properties (including mechanical strength, elongation, and pore size range) of the SAN nanofiber membranes for MD applications. The effect of solvent exposure time at a constant temperature on the final characteristics through the vapor welding process was investigated.

In the dilute solvent welding process, simple air-assisted spraying (instead of using electrospraying) was used to investigate the effect of spraying time and welding solution injection rate as these parameters were not mentioned in the previous study [33]. The time-consuming vapor welding process turned into a more time-efficient one by adjusting system parameters of the electroblowing process by using a mixed solvent system. The effects of changes in physical characteristics of the SAN membranes by various post-treatments on their desalination performance in a direct contact membrane distillation (DCMD) were assessed.

## 2. Materials and Methods

### 2.1. Materials

Commercial Grade SAN polymer (SAN-4) was purchased from Ghaed Bassir Petrochemical Products Co., Iran. DMSO, acetone, NaCl, isopropyl alcohol (IPA), and dimethylformamide (DMF) were all purchased from Ameretat Shimi (TAT Chem, Tehran, Iran). Cetyltrimethylammonium bromide (CTAB) salt was bought from Merck (Darmstadt, Germany) and used in the polymer spinning solution preparation.

### 2.2. Electroblowing Process

SAN nanofiber membranes were fabricated by the electroblowing process as described in our previous work [39]. In brief, the used spinning solution was composed of SAN/DMSO/acetone. 17.5 wt.% of SAN granules were added to the mixed solvent (DMSO to acetone volumetric ratio was 7:3) and stirred for 24 h under continuous heating of 60 °C. Additionally, a small amount of CTAB (0.25 wt.%) was added to the spinning solution. The degassed solution was then transferred to a plastic syringe. The spinning parameters for the fabrication of the neat SAN nanofiber membrane were illustrated in Table 1. The non-woven polypropylene (PP) was used as the substrate. To increase the concentration of the fabricated SAN nanofibers, both sides of the PP substrate were isolated using a wide tape (5 cm). This action enhances the thickness of the fabricated membranes and minimizes the inaccuracies through the width of the support. It needs to state that the PP substrate layer was removed before conducting the post-treatment processes.

### 2.3. Cold-/Hot-Pressing Processes

Cold-/hot-pressing processes were conducted to study the effects of pressing time and temperature with constant pressing pressure (2000 Psi) using a pressing device (GOTECH, Taichung city, Taiwan) on the final properties of SAN nanofiber membranes and desalination performance in MD. The pressing time in the cold-/hot-pressing processes was set at 10, 20, and 30 s. The applied temperature was started at 75 °C and finished at 105 °C (i.e., 75, 85, 95, and 105 °C). For the sake of clarity, the effect of time (i.e., 10, 20, and 30 s) on the final characteristics of the SAN membranes was only investigated at 75 °C. The cold-pressed membranes were labeled as CP-xs, where “xs” is the pressing time in seconds. For the hot-pressed membranes, HP-x °C-ys code was used, where “x °C” indicates the temperature of the pressing process and ys shows the time duration in seconds. For instance, CP-10 s and HP-75 °C-30 s indicate the cold-pressed SAN membranes for 10 s and hot-pressed SAN membranes at 75 °C for 30 s, respectively.

### 2.4. Vapor Welding Process

A 500 mL beaker was filled with the DMF solvent (50 mL) and covered with a screen to hold nanofiber samples. Then, the solvent was heated for about 1 h to reach 45 °C using a digital oven (UF30, Memmert, Germany) before initiating the welding process. The membrane samples were horizontally placed on the screen, and the solvent exposure time (i.e., 2, 6, 10, and 14 min) was changed to assess the physical characteristics of the post-treated samples. The surface of the beaker was covered with aluminum foil in every test. The VW-x code was used to indicate vapor welded membranes using different time durations at constant heating of 45 °C. The samples were then kept under ambient conditions for about 3 days before characterization.

### 2.5. Dilute Solvent Welding Process

An IPA non-solvent (19 mL) was mixed with one mL DMF solvent, and a spraying process was conducted on the membrane samples using an air-assisted spraying process. The whole fibrous structure can be sufficiently welded as the IPA fully wets the hydrophobic SAN nanofibrous membranes. The effects of critical parameters such as spraying time, welding solution injection rate, and working distance (i.e., tip-to-collector distance) on the final characteristics of the SAN membranes were investigated. The pre-sprayed samples were heated using an oven for 2 min. Additionally, the working distance was maintained at 10 or 15 cm during the spraying process. Welded membranes using dilute solvent were labeled as DS-x-y-z, where “x”, “y”, and “z” indicate the welding solution injection rate (mL/h), spraying time (min), and working distance (cm), respectively. Working distance was only mentioned for the samples welded at 10 cm. The spraying time was 2, 6, 10, 14, and 18 min for the samples treated at 40 mL/h injection rate and 15 cm working distance. Spraying time was also tested at 2, 6, and 10 min for the samples with 60 mL/h injection rate for 15 cm working distance or 40 mL/h injection rate for 10 cm working distance.

### 2.6. Characterization of SAN Nanofiber Membranes

The morphology observation was conducted by a scanning electron microscope (SEM) (Seron technology, Gyeonggi-do, Korea). Membrane samples were coated with gold before the SEM test. The fiber diameter of the fabricated samples was measured using Digimizer software, and an average of 100 measurements was reported. The membrane surface pore size was measured using the same software; then, the mean and maximum pore size was reported.

The porosity of the SAN samples was measured before and after soaking in IPA, and the pre-measured samples were weighed before and after wetting. The full description of the applied method can be found in the literature [40]. Membrane thickness was measured using an accurate digital micrometer and five measurements were conducted and mean value reported.

A custom-made set-up was used to measure the liquid entry pressure (LEP) of water. The samples were fastened between two semi-cells, and by helping the pressure of nitrogen, the pressure of the deionized (DI) water was increased step by step. The pressure where the water droplets were firstly seen on the membrane surface is regarded as the LEP value. The tests were repeated three times to ensure their reproducibility.

The water contact angle (WCA) of SAN membranes was assessed using a drop shape analyzer (KRUSS analyzer-G10 Drop Shape Analyzer, Germany). Glass slides were used to fix the sample on the holder, and 2 µL DI water was gently placed on the surface. The average value of three tests was then reported.

The mechanical properties were evaluated by a tensile testing device (Model 5566, Intron dynamometer, Buckinghamshire, UK). The load and stretching speed were 50 N and 5 mm/min, respectively. To conduct the tests accurately, the samples were strengthened with two rectangular cardboard frames, and the frame was scissored from both sides before the test.

### 2.7. A Bench-Scale DCMD Process

Desalination performance was assessed by using a bench-scale DCMD setup [41]. Shortly, the feed side was operated at temperature and feed flow rate of 60 °C and 0.48 L/min, respectively. For the permeate side, these values were 20 °C and 0.24 L/min, respectively. Synthetic seawater with 35 g/L NaCl was used for membrane assessment. The membrane-active area and module depth were 12.5 cm^2^ and 1 mm, respectively. The flow configuration was countercurrent. The electrical conductivity (EC) of the permeate tank was periodically monitored using an EC device (Metrohm-912, Novin Ebtekar, Tehran, Iran). The permeate flux (kg/m^2^ h) of the used samples was measured using the amount of added water into the permeate tank (kg) normalized by the effective area (m^2^) and time (h).

## 3. Results

### 3.1. Surface Morphology of SAN Nanofiber Membranes before and after Various Post-Treatment Methods

**Figure 1:**
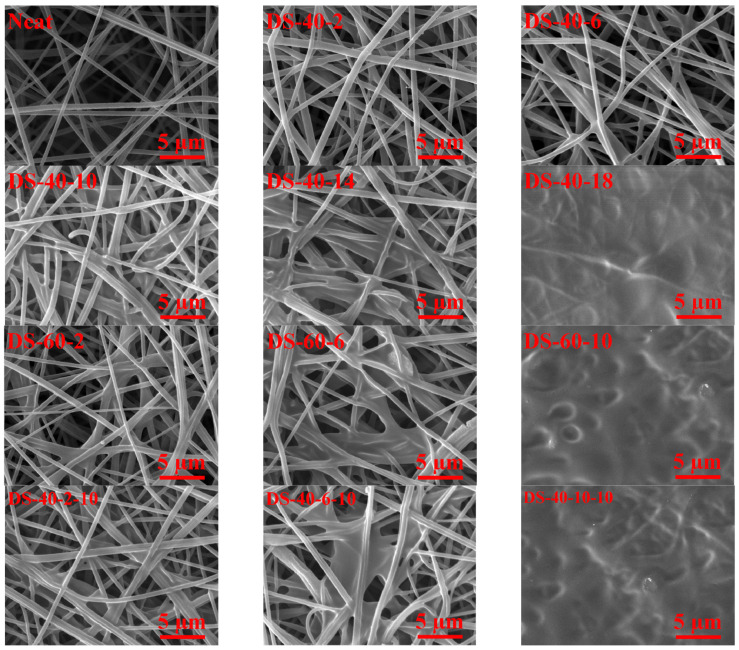
Surface morphology of SAN nanofiber membranes modified using the dilute solvent welding process. It is worth noting that the heating time was 2 min in all images.

The effects of spraying rate, tip-to-collector distance, and spraying time on the final morphology of the SAN nanofiber membranes were investigated. Figure 1 shows the SEM images of the SAN nanofiber membranes modified using the dilute welding process under different treatment conditions. Using 40 or 60 mL/h injection rates, the amount of welded fibers showed an increasing trend, as the time increased, and reached the point where the surface nanofibers fused to create a film-like surface without any void.

As shown in Figure 1, it seems that the 6 and 10 min for 40 mL/h injection rate and 2 min for 60 mL/h injection rate had proper morphology without excessive fiber welding. Moreover, by reducing tip-to-collector distance from 15 to 10 cm, the welding rate was increased considerably since the amount of deposited solution increased by reducing the spraying distance. As discussed in Section 3.4. Mechanical Properties of SAN Nanofiber Membranes, welding fibers in their junctions bestow considerable robustness to the whole fibrous web. Pore size reduction was obvious in all SEM images after conducting the dilute welding process, enhancing membrane wetting resistance better. Additionally, the mean fiber diameter of the neat samples increased gradually from 463 ± 51 nm to a maximum value of 670 ± 78 nm.

**Figure 2:**
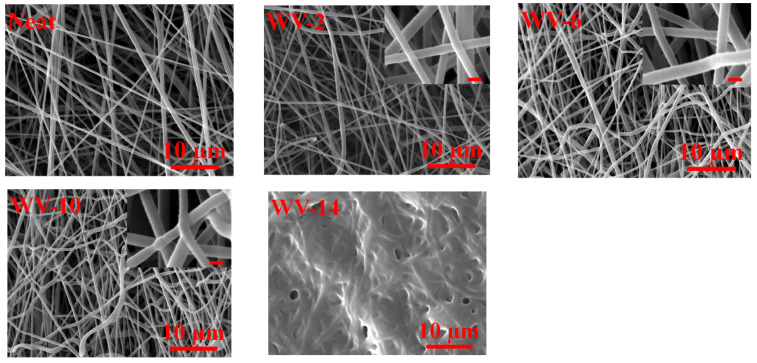
Surface morphology of SAN nanofiber membranes modified by the vapor welding process for 2–14 min at 45 °C. The inset images scale bar was 500 nm.

Figure 2 demonstrates the SEM images of the SAN nanofiber membranes treated by the vapor welding process at various solvent vapor exposure times. The temperature of the beakers was maintained at 45 °C during the tests. The exposure time is of great importance to increasing inter-fiber welding. Partial welding of 6 min exposure time turned into complete welding after 10 min exposure time (see inset images). After a 14 min vapor welding process, the surface of fibers was blocked entirely due to continuous contact with solvent vapor. Fiber diameter enhancement was negligible for the vapor welded nanofibers (from 2 to 10 min), as the solvent vapor in the reported welding times was not considerably softened fiber surfaces. Additionally, it should be noted that as DMSO solvent was used to fabricate neat SAN nanofibers, it is reasonable to conclude that a negligible amount of it can remain inside the fibrous network, but it is not high enough to automatically weld fibers.

**Figure 3:**
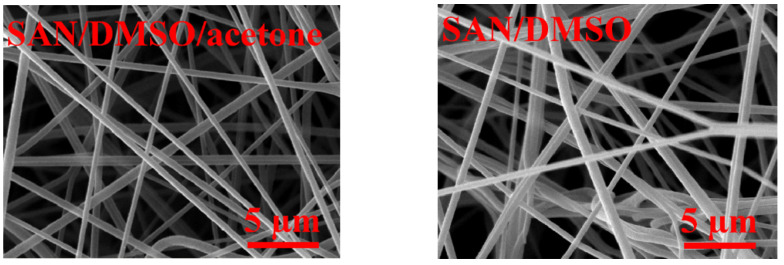
Surface morphology of the SAN nanofiber membranes fabricated with DMSO and mixed DMSO/acetone solvents.

Nanofibrous SAN membranes were fabricated using DSMO solvent and DMSO/acetone solvents (7:3 volumetric ratio). As shown in Figure 3, using a single DMSO solvent caused fiber fusion due to its excessive remaining. However, using the mixed solvent system, this undesirable effect did not occur due to the aid of acetone by reducing the boiling point of DMSO/acetone solvents (DMSO boiling point, 189 °C; acetone boiling point, 56 °C). The SAN/DMSO smelled as solvent even after 1 month, but SAN/DMSO/acetone solvent smelled less, and the solvent odor stopped after about 3 days of placing in the ambient condition. With neat membranes, the solvent exposure time was reduced using the simultaneous effect of remaining solvent and hot solvent vapors as the neat fabricated samples were directly applied to the vapor welding process with no delay.

**Figure 4:**
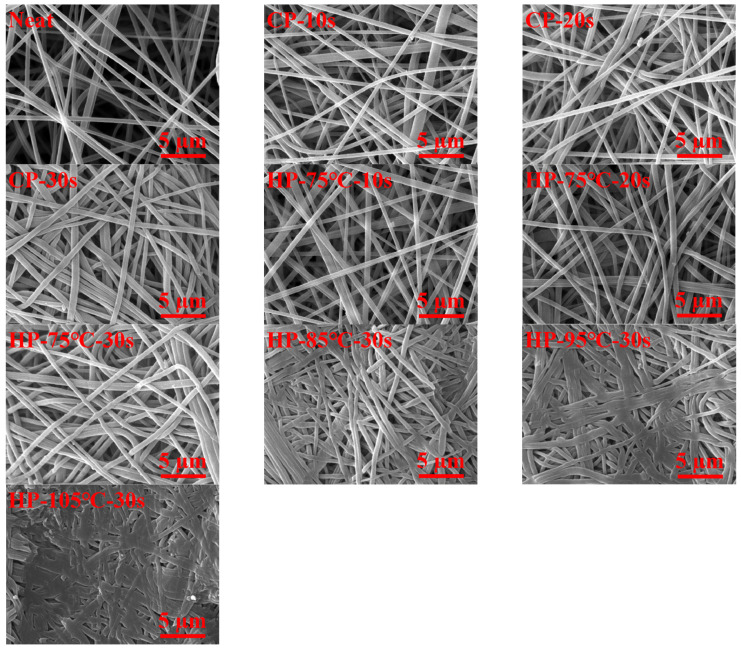
Surface morphology of SAN nanofiber membranes modified by the cold-/hot-pressing processes. Pressing pressure was maintained at 2000 Psi in all cases.

SEM images of the cold- and hot-pressed SAN nanofiber membrane samples were provided in Figure 4. Similar to the previous post-treatment processes, the cold-/hot-pressing processes effectively packed the nanofibrous structure to lessen pore size. Considering the cold-pressing process, the fiber diameter of the neat and post-treated samples did not show a significant difference. However, especially for the hot-pressed samples under a temperature of higher than 75 °C, fiber diameter was progressively increased up to 53%. This brought about a severe reduction in membrane porosity, pore size, thickness, and flexibility (see Table 2).

### 3.2. Porosity and Thickness of SAN Nanofiber Membranes

Higher porosity bestows high water flux and low heat conduction loss through the membrane length. Due to significant charge repulsion during the electroblowing process, high spinning rate, and using non-conductive PP support layer, the neat membrane structure tends to be looser than that of the post-treated membranes [42].

As shown in Table 2, The porosity of the membranes went under dilute welding process reduced from 96.2 to the minimum value of 57.6% by increasing the effect of fiber welding. This considerable reduction in porosity resulted from more severe fiber fusion. The minimum measured porosity value was 20.6% for the HP-105 °C-30 s membrane. It is worth mentioning that cold-pressed membranes showed higher porosity than those of hot-pressed ones, as the incorporation of heat compacts nanofibrous structure further.

Membrane thickness has an important role in determining membrane permeability and mechanical robustness. Thinner membranes enjoy having higher water flux and lower thermal conduction loss, whereas thicker ones can suppress thermal loss and negatively lower the water vapor permeation. The neat SAN membrane thickness experienced considerable reduction by imposing harsher conditions in all post-treatment processes. Among the data tabulated in Table 2, thickness reduction is the most pronounced phenomenon for the post-treated membranes. Therefore, it makes membranes more robust than the neat membrane, as discussed step by step in the following sections.

### 3.3. LEP Value

LEP is the factor to predict the wetting tendency of a hydrophobic membrane having a close relationship with surface hydrophobicity and maximum pore size. Neat nanofibrous membranes, due to non-woven and non-interlocked structures, are susceptible to pore deformation, larger maximum pore size, and, consequently, pore wetting. Additionally, the thickness of electrospun nanofiber membranes significantly affects the final pore size, as higher thicknesses have higher porosity and looser fibrous structure. It is therefore mandatory to decrease membrane pore size by employing different strategies.

The mean pore size of the neat SAN membranes showed the highest value with wider pore size distribution (see Figure 4). Using the dilute solvent welding process, the mean pore size was reduced from 1.63 µm, for the neat SAN membrane, to the minimum value of 0.54 µm, for the DS-40-14 membrane. Maximum pore size was also considerably reduced from 3.68, for the neat membrane, to 1.04 µm, for the DS-40-10.

In the vapor welding process, the mean and maximum pore size of VW-10 represented 2.39- and 2.06-times reduction, respectively. Considering the effect of the cold-pressing process on the post-treated membranes’ pore size, the highest reduction in pore size was attributable to the CP-30 s membrane.

**Figure 5:**
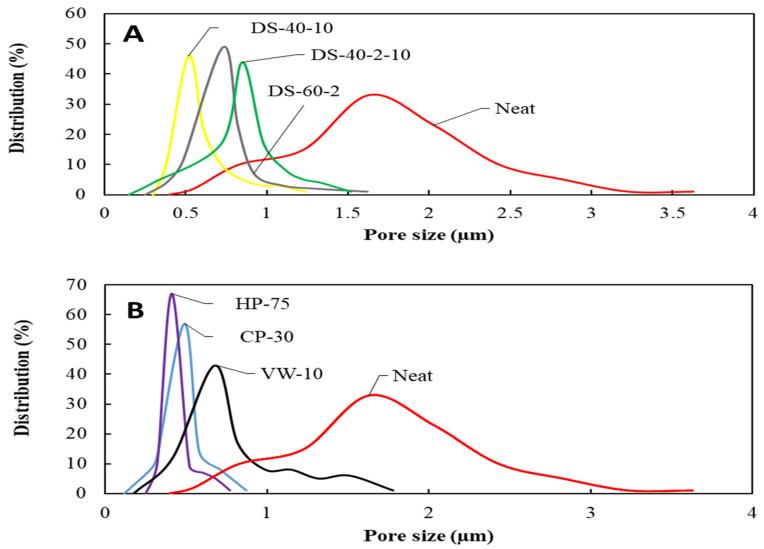
Pore size distribution of the neat SAN nanofiber membrane and membranes modified by the dilute solvent welding (**A**), vapor welding, and cold/hot pressing (**B**).

As shown in Table 2 previously, the mean and maximum pore size was reduced 3.32 and 4.22 times, compared with the neat membrane, showing the effect of proper pressing time and pressure on the pore size reduction. Additionally, by increasing the pressing process temperature from 75 to 105 °C, the fiber compaction improved to the point where the surface pore size and porosity disappeared. Additionally, as shown in Figure 5, the pore size distribution of the post-treated membranes became more centralized than that of the neat membrane; therefore, the risk of pore wetting diminished significantly.

The WCA of the used membranes is tabulated in Table 3. A gradual reduction in WCA was observed for the post-treated membranes. For example, the WCA of the DS-40-10 membrane was measured as 134.2°, showing a 7.89% lower WCA than the neat membrane (145.7°). This was due to the gradual decline in surface roughness by progressing the fiber fusion and compaction [26,32]. WCA decline for the HP-105 °C-30 s was more evident as the fibrous structure turned into a film-like surface. By increasing welding solution feeding rate, feeding time, and reducing working distance, the welded spot grows undesirably to block the water vapor passage and reduce porosity. Only DS-40-10 and DS-40-14 membranes from the dilute solvent welding category were applied to the DCMD process. The VW-10, CP-30 s, HP-75 °C-30 s, and HP-85 °C-30 s membranes were also used for salty water desalination due to better LEP value, lower thickness, and reasonable porosity.

### 3.4. Mechanical Properties of SAN Nanofiber Membranes

Tensile strength and strain of prepared SAN membranes were measured and presented in Table 3. All membranes displayed better mechanical characteristics than the neat ones. The dilute welded DS-40-10 membrane showed the highest elongation at a break of 33.28%. However, the increasing welding reduced membrane flexibility due to detrimental fiber fusion, especially for the DS-60-6 membrane. The tensile strength was further increased from 1.32 MPa for the neat nanofibrous SAN membrane to 5.87 MPa for the DS-60-6 membrane. The substantial improvement in tensile strength was due to solvent-induced fiber fusion.

Using the vapor welding process, a similar enhancement in tensile strength and strain at break was observed. Cold-pressed membranes exhibited even more reinforcement in tensile strength than those of dilute solvent welding and vapor welding processes. Hot-pressed membranes were superior in the case of mechanical robustness. The application of thermal pressing made the neat SAN membranes much more robust than the other post-treated membrane, regardless of higher loss in membrane flexibility. However, a temperature higher than 85 °C is not suggested since WCA, porosity, and membrane flexibility were severely sacrificed to fabricate a rigid membrane.

### 3.5. Performance of the Bench-Scale DCMD Process with SAN Nanofiber Membranes

**Figure 6:**
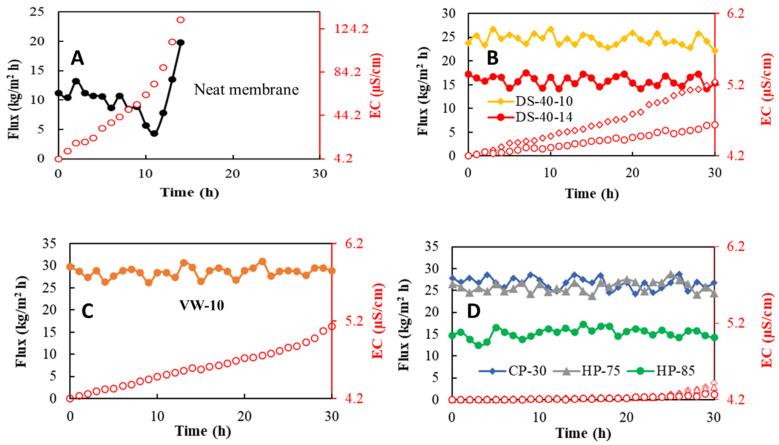
Water vapor flux and permeate EC of the neat (**A**), DS-40-10 and DS-40-14 (**B**), VW-10 (**C**), and CP-30 s, HP-75 °C-30 s and HP-85 °C-30 s (**D**) membranes in the DCMD process (feed concentration = 35 g/L NaCl; temperature difference = 40 °C; feed flow rate = 0.48 L/min; permeate flow rate = 0.48 L/min). Red hollow symbols illustrate EC of membrane distillate.

The optimum SAN membranes of each category were used for the desalination of synthetic seawater (35 g/L NaCl) in the bench-scale DCMD process. The flux–time diagrams are presented in Figure 6. The salt rejection factor and final EC of the used membranes are arranged in Table 4. The permeate flux followed the order of VW-10 > CP-30 s > HP-75 °C-30 s > DS-40-10 > DS-40-14 > HP-85 °C-30 s > neat. Despite having the highest porosity and pore size, neat nanofibrous SAN membranes demonstrated the weakest DCMD desalination performance due to the highest measured thickness and lowest LEP value.

The best permeable membrane was the VW-10 because of the highest porosity and reasonably bigger mean pore size. Although it is possible to adjust vapor welding conditions to increase the intensity of solvent vapor induced welding, a recent study conducted by Su et al. [43] showed that it could diminish porosity from 83 to 42%. The meaningful reduction in permeability of DS-40-14 regardless of having lower thickness than DS-40-10 was due to pore blockage made by severer surface welding. The CP-30 s and HP-75 °C-30 s membranes exhibited proper desalination performance because of higher LEP value, except for the HP-85 °C-30 s membrane. The lowest porosity and pore size, and lower WCA were the main reasons for lower water permeability.

## 4. Conclusions

Neat SAN nanofiber membranes were fabricated using the electroblowing process, and then various post-treatment processes were applied to enhance the mechanical properties of the SAN membrane for MD applications. The main results of this work were summarized as follows:

- Neat SAN membrane lacked suitable mechanical strength and wetting resistance for MD applications despite having higher porosity, pore size, and hydrophobicity;
- In the dilute solvent welding process, solution injection rate and tip-to-collector distance have the dominant role in forming the final morphology of SAN membranes;
- The vapor welding process is a suitable option to reinforce nanofiber membranes by keeping high porosity;
- Cold-/hot-pressing processes exhibited the best in reducing pore size and increasing the robustness of the whole nanofiber structure;
- WCA reduction is not avoidable in all of the processes because of surface roughness reduction, and it has a direct relation with the degree of post-treatment;
- In the dilute solvent and vapor welding processes, the nanofiber web can be more flexible to some extent; however, in pressing processes, membrane flexibility gives way to a more robust membrane;
- Pore size and its distribution were reduced appropriately after post-treatment processes to create anti-deformable/wetting membranes;
- A bench-scale DCMD process with the modified SAN nanofiber membranes showed stable salt rejection (>99.9% removal of salts) and permeate flux for 30 h operation without membrane wetting.

## Figures and Tables

**Table 1:** Voltage, polymer injection rate, working distance, air flow rate, rotating speed, and spinning time for the fabrication of neat SAN membranes.

Membrane	Voltage (kV)	Injection Rate (µL/min)	Working Distance (cm)	Airflow Rate (NL/min)	Rotating Speed (rpm)	Spinning Time (min)
Neat SAN	18	90	30	4	250	60

**Table 2:** Thickness (δ), porosity (ε), and pore size of the post-treated SAN nanofiber membranes.

Post-Treatment	Sample Name	δ (µm)	ε (%)	r_mean_ (µm)	r_max_ (µm)
Dilute solvent welding	Neat	845 ± 40	96.2 ± 2	1.63 ± 0.02	3.68 ± 0.05
	DS-40-2	710 ± 20	92.2 ± 2	0.81 ± 0.02	2.11 ± 0.04
	DS-40-6	570 ± 25	89.2 ± 2	0.76 ± 0.02	1.67 ± 0.04
	DS-40-10	320 ± 10	73.2 ± 2	0.61 ± 0.01	1.24 ± 0.03
	DS-40-14	210 ± 5	62.1 ± 2	0.54 ± 0.01	1.04 ± 0.02
	DS-40-2-10	430 ± 4	82.5 ± 2	0.72 ± 0.02	1.52 ± 0.04
	DS-40-6-10	205 ± 4	62.6 ± 2	0.63 ± 0.01	1.45 ± 0.03
	DS-60-2	420 ± 10	82.4 ± 2	0.73 ± 0.02	1.62 ± 0.04
	DS-60-6	180 ± 3	57.6 ± 2	0.58 ± 0.03	1.23 ± 0.03
Vapor welding	VW-2	820 ± 30	95.1 ± 2	0.82 ± 0.03	3.42 ± 0.05
	VW-6	510 ± 15	86.3 ± 2	0.74 ± 0.02	1.53 ± 0.04
	VW-10	250 ± 3	79.1 ± 2	0.68 ± 0.02	1.78 ± 0.04
Cold/hot pressing	CP-10 s	192 ± 4	74.2 ± 1	0.56 ± 0.01	1.11 ± 0.02
	CP-20 s	171 ± 4	72.2 ± 1	0.53 ± 0.01	0.92 ± 0.02
	CP-30 s	152 ± 2	69.1 ± 1	0.49 ± 0.01	0.87 ± 0.02
	HP-75 °C-10 s	178 ± 3	72.1 ± 1	0.54 ± 0.01	0.91 ± 0.01
	HP-75 °C-20 s	157 ± 2	70.4 ± 1	0.51 ± 0.01	0.88 ± 0.01
	HP-75 °C-30 s	132 ± 2	65.1 ± 2	0.41 ± 0.02	0.77 ± 0.02
	HP-85 °C-30 s	106 ± 2	46.3 ± 2	0.33 ± 0.01	0.64 ± 0.01
	HP-95 °C-30 s	85 ± 1	32.5 ± 1	0.24 ± 0.01	0.51 ± 0.01
	HP-105 °C-30 s	61 ± 1	18.6 ± 1	-	-

**Table 3:** WCA, LEP value, and mechanical properties of the SAN nanofiber membranes modified by various post-treatment methods.

Post-Treatment	Sample Name	WCA (°)	LEP (kPa)	Tensile Strength (MPa)	Strain (%)
Dilute solvent welding	Neat	145.7 ± 1	40.8 ± 2	1.32 ± 0.2	25.21 ± 0.7
	DS-40-2	143.7 ± 1	61.3 ± 2	1.98 ± 0.3	27.98 ± 0.4
	DS-40-6	140.1 ± 1	73.2 ± 2	2.85 ± 0.2	29.45 ± 0.4
	DS-40-10	134.2 ± 1	83.3 ±2	3.94 ± 0.4	33.28 ± 0.3
	DS-40-14	124.3 ± 1	99.7 ± 2	4.68 ± 0.3	20.32 ± 0.8
	DS-40-2-10	137.9 ± 1	77.5 ± 2	3.25 ± 0.2	28.96 ± 0.7
	DS-40-6-10	125.2 ± 1	73.6 ± 2	5.23 ± 0.4	19.12 ± 0.3
	DS-60-2	135.3 ± 1	76.4 ± 2	3.12 ± 0.4	30.23 ± 0.6
	DS-60-6	123.6 ± 1	91.8 ± 2	5.87 ± 0.5	16.78 ± 0.3
Vapor welding	VW-2	144.7 ± 1	54.2 ± 2	1.43 ± 0.3	26.32 ± 0.5
	VW-6	142.1 ± 1	80.4 ± 2	2.87 ± 0.3	27.96 ± 0.4
	VW-10	139.2 ± 1	87.6 ± 2	3.96 ± 0.2	31.24 ± 1
Cold/hot pressing	CP-10s	141.3 ± 1	103.9 ± 1	6.14 ± 0.4	23.14 ± 0.6
	CP-20s	139.8 ± 1	112.8 ± 1	6.97 ± 0.5	21.12 ± 0.7
	CP-30s	137.2 ± 1	123.4 ± 1	7.86 ± 0.2	18.42 ± 0.9
	HP-75 °C-10 s	142.1 ± 1	116.7 ± 1	6.57 ± 0.6	21.74 ± 0.9
	HP-75 °C-20 s	138.4 ± 1	127.5 ± 1	7.63 ± 0.3	19.23 ± 1
	HP-75 °C-30 s	134.2 ± 1	138.1 ± 2	9.23 ± 0.3	16.75 ± 1
	HP-85 °C-30 s	120.2 ± 1	147.1 ± 2	10.24 ± 0.4	11.23 ± 1
	HP-95 °C-30 s	112.4 ± 1	159.2 ± 1	12.24 ± 0.2	6.45 ± 1
	HP-105 °C-30 s	93.1 ± 1	-	14.68 ± 0.1	3.16 ± 0.5

**Table 4:** Permeate flux, final EC of the permeate, and salt rejection factor for the employed SAN membranes in the bench-scale DCMD process. Initial permeate conductivity was 4.2 µS/cm.

Membrane	Permeate Flux (kg/m^2^ h)	Final EC (µS/cm)	Salt Rejection Factor (%)	Duration (h)
Neat	10.34	132.74	>98	14
DS-40-10	24.46	5.24	>99.9	30
DS-40-14	15.86	4.64	>99.9	30
VW-10	28.60	5.13	>99.9	30
CP-30s	26.68	4.42	>99.9	30
HP-75 °C-30 s	25.81	4.37	>99.9	30
HP-85 °C-30 s	15.24	4.27	>99.9	30

## References

[1] Xie B., Xu G., Jia Y., Gu L., Wang Q., Mushtaq N., Cheng B., Hu Y. (2021). Engineering carbon nanotubes enhanced hydrophobic membranes with high performance in membrane distillation by spray coating. J. Membr. Sci..

[2] Grasso G., Galiano F., Yoo M.J., Mancuso R., Park H.B., Gabriele B., Figoli A., Drioli E. (2020). Development of graphene-PVDF composite membranes for membrane distillation. J. Membr. Sci..

[3] Zhu Z., Zhong L., Horseman T., Liu Z., Zeng G., Li Z., Lin S., Wang W. (2021). Superhydrophobic-omniphobic membrane with anti-deformable pores for membrane distillation with excellent wetting resistance. J. Membr. Sci..

[4] Alkhudhiri A., Darwish N., Hilal N. (2012). Membrane distillation: A comprehensive review. Desalination.

[5] Chiam C.-K., Sarbatly R. (2013). Vacuum membrane distillation processes for aqueous solution treatment-A review. Chem. Eng. Process. Process Intensif..

[6] Li X., Yu X., Cheng C., Deng L., Wang M., Wang X. (2015). Electrospun superhydrophobic organic/inorganic composite nanofibrous membranes for membrane distillation. ACS Appl. Mater. Interfaces.

[7] Qing W., Wu Y., Li X., Shi X., Shao S., Mei Y., Zhang W., Tang C.Y. (2020). Omniphobic PVDF nanofibrous membrane for superior anti-wetting performance in direct contact membrane distillation. J. Membr. Sci..

[8] Tijing L.D., Woo Y.C., Choi J.-S., Lee S., Kim S.-H., Shon H.K. (2015). Fouling and its control in membrane distillation—A review. J. Membr. Sci..

[9] Tian R., Gao H., Yang X.H., Yan S.Y., Li S. (2014). A new enhancement technique on air gap membrane distillation. Desalination.

[10] Geng H., He Q., Wu H., Li P., Zhang C., Chang H. (2014). Experimental study of hollow fiber AGMD modules with energy recovery for high saline water desalination. Desalination.

[11] Alkhudhiri A., Darwish N., Hilal N. (2013). Treatment of saline solutions using air gap membrane distillation: Experimental study. Desalination.

[12] Fan H., Peng Y. (2012). Application of PVDF membranes in desalination and comparison of the VMD and DCMD processes. Chem. Eng. Sci..

[13] Dong Z.-Q., Ma X., Xu Z.-L., You W.-T., Li F. (2014). Superhydrophobic PVDF-PTFE electrospun nanofibrous membranes for desalination by vacuum membrane distillation. Desalination.

[14] Zhang P., Knötig P., Gray S., Duke M. (2015). Scale reduction and cleaning techniques during direct contact membrane distillation of seawater reverse osmosis brine. Desalination.

[15] Lee E.-J., An A.K., He T., Woo Y.C., Shon H.K. (2016). Electrospun nanofiber membranes incorporating fluorosilane-coated TiO_2_ nanocomposite for direct contact membrane distillation. J. Membr. Sci..

[16] Kayvani Fard A., Rhadfi T., Khraisheh M., Atieh M.A., Khraisheh M., Hilal N. (2016). Reducing flux decline and fouling of direct contact membrane distillation by utilizing thermal brine from MSF desalination plant. Desalination.

[17] Francis L., Ghaffour N., Alsaadi A.S., Nunes S.P., Amy G.L. (2014). Performance evaluation of the DCMD desalination process under bench scale and large scale module operating conditions. J. Membr. Sci..

[18] Ge J., Peng Y., Li Z., Chen P., Wang S. (2014). Membrane fouling and wetting in a DCMD process for RO brine concentration. Desalination.

[19] Song Z.W., Jiang L.Y. (2013). Optimization of morphology and performance of PVDF hollow fiber for direct contact membrane distillation using experimental design. Chem. Eng. Sci..

[20] Nghiem L.D., Cath T. (2011). A scaling mitigation approach during direct contact membrane distillation. Sep. Purif. Technol..

[21] Seyed Shahabadi S.M., Rabiee H., Seyedi S.M., Mokhtare A., Brant J.A. (2017). Superhydrophobic dual layer functionalized titanium dioxide/polyvinylidene fluoride-co-hexafluoropropylene (TiO_2_/PH) nanofibrous membrane for high flux membrane distillation. J. Membr. Sci..

[22] Liao Y., Loh C.H., Tian M., Wang R., Fane A.G. (2018). Progress in electrospun polymeric nanofibrous membranes for water treatment: Fabrication, modification and applications. Prog. Polym. Sci..

[23] Tijing L.D., Choi J.-S., Lee S., Kim S.-H., Shon H.K. (2014). Recent progress of membrane distillation using electrospun nanofibrous membrane. J. Membr. Sci..

[24] Feng C., Khulbe K.C., Matsuura T., Tabe S., Ismail A.F. (2013). Preparation and characterization of electro-spun nanofiber membranes and their possible applications in water treatment. Sep. Purif. Technol..

[25] Namsaeng J., Punyodom W., Worajittiphon P. (2019). Synergistic effect of welding electrospun fibers and MWCNT reinforcement on strength enhancement of PAN–PVC non-woven mats for water filtration. Chem. Eng. Sci..

[26] Zhao Z., Zheng J., Wang M., Zhang H., Han C.C. (2012). High performance ultrafiltration membrane based on modified chitosan coating and electrospun nanofibrous PVDF scaffolds. J. Membr. Sci..

[27] Yao M., Woo Y.C., Tijing L.D., Shim W.-G., Choi J.-S., Kim S.-H., Shon H.K. (2016). Effect of heat-press conditions on electrospun membranes for desalination by direct contact membrane distillation. Desalination.

[28] Na H., Zhao Y., Zhao C., Zhao C., Yuan X. (2008). Effect of hot-press on electrospun poly (vinylidene fluoride) membranes. Polym. Eng. Sci..

[29] Wang W., Jin X., Zhu Y., Zhu C., Yang J., Wang H., Lin T. (2016). Effect of vapor-phase glutaraldehyde crosslinking on electrospun starch fibers. Carbohydr. Polym..

[30] Shaulsky E., Nejati S., Boo C., Perreault F., Osuji C.O., Elimelech M. (2017). Post-fabrication modification of electrospun nanofiber mats with polymer coating for membrane distillation applications. J. Membr. Sci..

[31] Deng L., Li P., Liu K., Wang X., Hsiao B.S. (2019). Robust superhydrophobic dual layer nanofibrous composite membranes with a hierarchically structured amorphous polypropylene skin for membrane distillation. J. Mater. Chem. A.

[32] Huang L., Manickam S.S., McCutcheon J.R. (2013). Increasing strength of electrospun nanofiber membranes for water filtration using solvent vapor. J. Membr. Sci..

[33] Su C., Lu C., Horseman T., Cao H., Duan F., Li L., Li M., Li Y. (2020). Dilute solvent welding: A quick and scalable approach for enhancing the mechanical properties and narrowing the pore size distribution of electrospun nanofibrous membrane. J. Membr. Sci..

[34] Rianjanu A., Kusumaatmaja A., Suyono E.A., Triyana K. (2018). Solvent vapor treatment improves mechanical strength of electrospun polyvinyl alcohol nanofibers. Heliyon.

[35] Sazegar M., Bazgir S., Katbab A.A. (2020). Preparation and characterization of water-absorbing gas-assisted electrospun nanofibers based on poly(vinyl alcohol)/chitosan. Mater. Today Commun..

[36] Niknejad A.S., Bazgir S., Ardjmand M., Shirazi M.M.A. (2020). Spent caustic wastewater treatment using direct contact membrane distillation with electroblown styrene-acrylonitrile membrane. Int. J. Environ. Sci. Technol..

[37] Niknejad A.S., Bazgir S., Sadeghzadeh A., Shirazi M.M.A. (2020). Styrene-acrylonitrile (SAN) nanofibrous membranes with unique properties for desalination by direct contact membrane distillation (DCMD) process. Desalination.

[38] Khoshnevisan S., Bazgir S. (2020). Treatment of dye wastewater by direct contact membrane distillation using superhydrophobic nanofibrous high-impact polystyrene membranes. Int. J. Environ. Sci. Technol..

[39] Sadeghzadeh A., Bazgir S., Shirazi M.M.A. (2020). Fabrication and characterization of a novel hydrophobic polystyrene membrane using electroblowing technique for desalination by direct contact membrane distillation. Sep. Purif. Technol..

[40] Li X., Wang C., Yang Y., Wang X., Zhu M., Hsiao B.S. (2014). Dual-biomimetic superhydrophobic electrospun polystyrene nanofibrous membranes for membrane distillation. ACS Appl. Mater. Interfaces.

[41] Niknejad A.S., Bazgir S., Kargari A. (2021). Mechanically improved superhydrophobic nanofibrous polystyrene/high-impact polystyrene membranes for promising membrane distillation application. J. Appl. Polym. Sci..

[42] Niknejad A.S., Bazgir S., Kargari A. (2021). Desalination by direct contact membrane distillation using a superhydrophobic nanofibrous poly (methyl methacrylate) membrane. Desalination.

[43] Su C., Lu C., Cao H., Tang K., Chang J., Duan F., Ma X., Li Y. (2018). Fabrication and post-treatment of nanofibers-covered hollow fiber membranes for membrane distillation. J. Membr. Sci..

